# Extension of Pharmacokinetic/Pharmacodynamic Time-Kill Studies To Include Lipopolysaccharide/Endotoxin Release from Escherichia coli Exposed to Cefuroxime

**DOI:** 10.1128/AAC.02070-19

**Published:** 2020-03-24

**Authors:** Anders Thorsted, Eva Tano, Kia Kaivonen, Jan Sjölin, Lena E. Friberg, Elisabet I. Nielsen

**Affiliations:** a Department of Pharmaceutical Biosciences, Uppsala University, Uppsala, Sweden; b Department of Medical Sciences, Uppsala University Hospital, Uppsala, Sweden

**Keywords:** LPS, pharmacokinetics/pharmacodynamics, cefuroxime, endotoxin, time-kill

## Abstract

The release of inflammatory bacterial products, such as lipopolysaccharide (LPS)/endotoxin, may be increased upon the administration of antibiotics. An improved quantitative understanding of endotoxin release and its relation to antibiotic exposure and bacterial growth/killing may be gained by an integrated analysis of these processes. The aim of this work was to establish a mathematical model that relates Escherichia coli growth/killing dynamics at various cefuroxime concentrations to endotoxin release *in vitro*. Fifty-two time-kill experiments informed bacterial and endotoxin time courses and included both static (0×, 0.5×, 1×, 2×, 10×, and 50× MIC) and dynamic (0×, 15×, and 30× MIC) cefuroxime concentrations. A model for the antibiotic-bacterium interaction was established, and antibiotic-induced bacterial killing followed a sigmoidal Emax relation to the cefuroxime concentration (MIC-specific 50% effective concentration [EC_50_], maximum antibiotic-induced killing rate [*E*_max_] = 3.26 h^−1^ and γ = 3.37). Endotoxin release was assessed in relation to the bacterial processes of growth, antibiotic-induced bacterial killing, and natural bacterial death and found to be quantitatively related to bacterial growth (0.000292 endotoxin units [EU]/CFU) and antibiotic-induced bacterial killing (0.00636 EU/CFU). Increased release following the administration of a second cefuroxime dose was described by the formation and subsequent antibiotic-induced killing of filaments (0.295 EU/CFU). Release due to growth was instantaneous, while release due to antibiotic-induced killing was delayed (mean transit time of 7.63 h). To conclude, the *in vitro* release of endotoxin is related to bacterial growth and antibiotic-induced killing, with higher rates of release upon the killing of formed filaments. Endotoxin release over 24 h is lowest when antibiotic exposure rapidly eradicates bacteria, while increased release is predicted to occur when growth and antibiotic-induced killing occur simultaneously.

## Abstract (precis)

The release of inflammatory bacterial products, such as lipopolysaccharide (LPS)/endotoxin, may be increased upon the administration of antibiotics. An improved quantitative understanding of endotoxin release and its relation to antibiotic exposure and bacterial growth/killing may be gained by an integrated analysis of these processes. The aim of this work was to establish a mathematical model that relates Escherichia coli growth/killing dynamics at various cefuroxime concentrations to endotoxin release *in vitro*.

## TEXT

Gram-negative bacterial infections constitute a serious health problem and are associated with high mortality rates in the intensive care unit (1, 2). A large portion of the outer plasma membrane of Gram-negative bacteria consists of lipopolysaccharide (LPS)/endotoxin (3, 4). Innate immune cell recognition of endotoxin will alert host defenses and establish an inflammatory response aimed at the infection, but this response might be dysregulated, leading to tissue and organ injury in the host (5). In piglets, the immune response has been shown to intensify with increased endotoxin exposure and/or rate of delivery (6, 7). However, while a correlation between endotoxemia and patient mortality has been shown in some studies (8, 9), the overall evidence is inconclusive (10, 11), and the prognostic value of endotoxemia remains unresolved (12).

According to guidelines, empirical intravenous administration of one or multiple broad-spectrum antibiotics should be initiated within 1 h of the recognition of sepsis to cover all likely pathogens (13). The mechanism of action for antibiotic-induced bacterial death directly influences endotoxin release (14), with β-lactams known to be potent inducers of release (15). The β-lactams target the penicillin-binding proteins (PBPs), important enzymes (transpeptidases) that catalyze cross-linking reactions during bacterial cell wall synthesis (16). As specificity for a single PBP is rare, antibiotic-induced killing is the result of combined PBP inhibition and differentiated effects across the concentration gradient (17, 18). PBPs 1 to 3 are of primary importance for bacterial survival, and as general Gram-negative examples, inhibition of Escherichia coli-specific PBPs 1 to 3 results in bacterial lysis, the formation of spheroplasts, and the formation of long filaments, respectively (19). Individual microbial growth without an increase in CFU (i.e., biomass increase due to filament formation) is observed for antibiotics that primarily bind PBP-3, such as the second-generation cephalosporin cefuroxime and antibiotics commonly used for the treatment of sepsis, such as piperacillin-tazobactam, cefotaxime, and meropenem (17, 19, 20). PBP-3 binding may lead to a large endotoxin release upon final bacterial lysis (10, 19), with filament formation found to generally occur at concentration ranges above the pathogen MIC (21). As PBP-1 binding (i.e., lysis) occurs at higher antibiotic concentrations than PBP-3 binding, extensive filament production might occur at clinically relevant concentrations for less susceptible microorganisms (17, 18, 21).

Mathematical models that simultaneously describe bacterial growth and antibiotic-induced killing over time have been developed and help to facilitate a quantitative understanding of antibiotic-bacterium interactions (22). Such models typically describe exponential bacterial growth up to a maximum capacity and account for antibiotic-induced killing and natural bacterial death (23). This quantification and separation of antibiotic-bacterial processes could be utilized in the quantification of endotoxin release. Quantitative models find additional value when used in prediction, for instance, of scenarios of interest to study further experimentally, for optimization of doses and/or sampling times, or for hypothesis generation through increased system understanding.

In this work, we aim to quantify the *in vitro* release of endotoxin from E. coli exposed to both static and dynamic concentrations of cefuroxime, a PBP-3-active β-lactam antibiotic. Processes accounting for bacterial dynamics (such as growth and antibiotic-induced kill) were linked to endotoxin release, resulting in an integrated mathematical model with a specific focus on the time course of endotoxin release, and release for a number of clinically used cefuroxime regimens was predicted.

## RESULTS

The mathematical framework was established based on newly generated static time-kill experiments (*n* = 28) and data from previously reported dynamic time-kill experiments (*n* = 24) (24).

**FIG 1:**
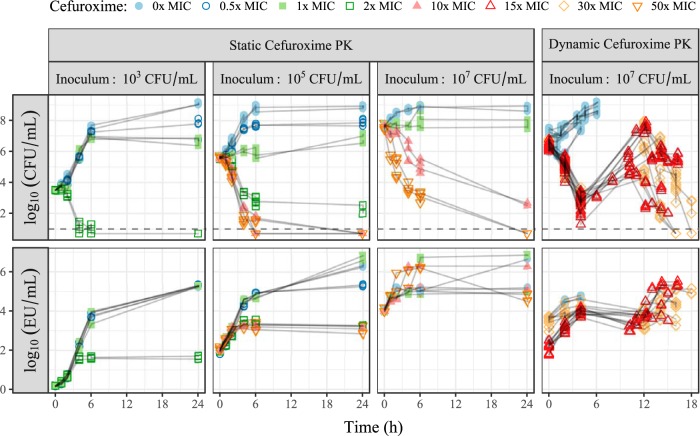
Overview of available data (points) from individual experiments (connected with lines), showing the time courses of CFU (top) and endotoxin units (EU) (bottom). The studies were performed with static or dynamic cefuroxime concentrations at various starting inocula, and the color and shape of the points refer to cefuroxime exposure, as indicated in the key. The horizontal dashed lines indicate the lower limit of detection (10 CFU/ml). PK, pharmacokinetics.

**FIG 2:**
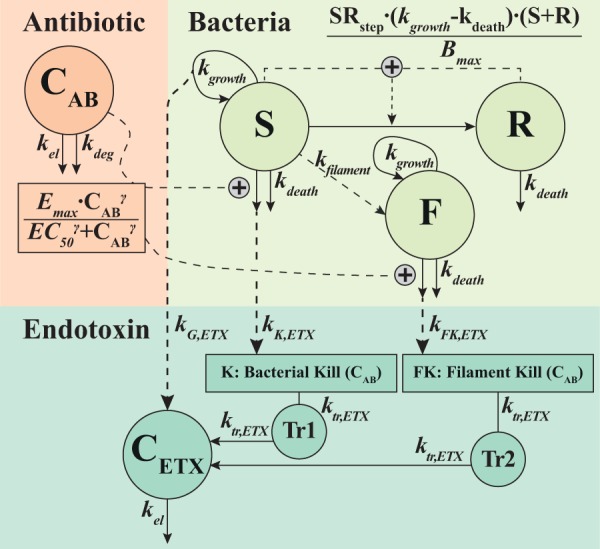
Schematic of the final model, linking the antibiotic concentration-time course (*C_AB_*) to the bacterial dynamics represented by the *S* (susceptible), *R* (resting), and *F* (filament) states, with the antibiotic stimulating killing of the *S* (and *F*) population(s) through a sigmoidal Emax relationship to cefuroxime exposure (*C_AB_*). The increase in the LPS/endotoxin concentration (*C_ETX_*) was driven by the growth of the bacteria in *S* (direct contribution to *C_ETX_*) and by the antibiotic-induced killing of bacteria in *S* and *F* (delayed contribution to *C_ETX_* through transit compartments 1 [Tr1] and 2 [Tr2]). See Table 1 for parameter estimates.

The antibiotic cefuroxime was one of the most commonly used antibiotics in Swedish hospitals at the time of the dynamic experiments (25) and was chosen for consistency in the generation of the new static data. Static study data contributed 382 CFU counts (8 below the limit of detection [LOD]; on average, 2.3 counts per time point) and 168 endotoxin concentrations (all quantified). Approximate observed start inocula were 3 × 10^3^, 4 × 10^5^, and 4.5 × 10^7^ CFU/ml, with data illustrated in Fig. 1. The expected relationship between antibiotic exposure and bacterial killing was observed, with 2× MIC needed for a bactericidal effect with a medium/low start inoculum and a reduced killing effect at a higher start inoculum. The endotoxin level increased approximately 1 log-fold at high drug exposures and significantly more in controls and 0.5× and 1× MIC experiments. The dynamic study data consisted of 439 counts of CFU (4 below the LOD; on average, 4 counts per time point) and 102 measurements of endotoxin (all quantified) with an approximate start inoculum of 2.5 × 10^6^ CFU/ml. Rapid initial killing was followed by significant variability in bacterial regrowth (around 12 h) and was mirrored in the time course of endotoxin. A schematic of the final model framework is presented in Fig. 2.

### Bacterial dynamics.

The bacterial dynamics were well characterized by the model structure previously described (23), with a few adaptations. The model fit improved significantly when estimating separate 50% effective concentration (EC_50_) values for MIC values of 2 and 4 mg/liter (difference in objective function value [ΔOFV] = −87.20), with estimates of 2.93 and 4.88 mg/liter. The transition from a susceptible state (*S*) to a resting bacterial state (*R*) was coupled to a step function, with an estimated threshold (*T_SR_*) of total bacteria (*S* + *R*), according to the following equation:

(1) $$SR_{\text{step}}=\frac{{\left(S+R\right)}^{10}}{{\left(S+R\right)}^{10}+T_{\mathrm{SR}}{}^{10}}$$

where the power of 10 produces an on-off relation and the value of *SR*_step_ (0 to 1) is multiplied on the transition function, as indicated in Fig. 2 (ΔOFV = −37.97). The high *T_SR_* estimate (7 × 10^7^ CFU/ml) keeps most bacteria in the susceptible state and reflects that rapid killing was initially observed in high-inoculum experiments. Finally, a mixture model with two populations (ΔOFV = −33.43) identified 43% of the dynamic experiments as having a 36% higher elimination rate (*k_el_*) for cefuroxime than the planned 0.426 h^−1^ (the remaining experiments had the intended *k_el_*).

### Endotoxin release with static cefuroxime concentrations.

An increase in the endotoxin concentration was observed across all experiments, including controls, indicating that release occurs during normal growth and antibiotic-induced bacterial killing. The statistically most significant effect, based on equations 5 to 7, was observed when binary fission (dividing bacteria) was driving the increase in endotoxin (*k_G_*_,_*_ETX_*, ΔOFV = −1,086.68), improving further with the addition of an effect from antibiotic-induced bacterial killing (*k_K_*_,_*_ETX_*, ΔOFV = −84.67). A contribution from natural bacterial death was significant (*k_D_*_,_*_ETX_*, ΔOFV = −7.67 [*P* < 0.01]) but was omitted due to high uncertainty in the parameter estimate.

### Endotoxin release with dynamic cefuroxime concentrations and filament formation.

While release following the first cefuroxime dose was well described by applying the model developed based on static data (with endotoxin eliminated at the rate *k_el_*), the increased release following the second dose could not be adequately captured. As the formation of filaments was the most likely mechanism, susceptible bacteria were allowed to transition to a filamentous state (*F*) at certain antibiotic concentrations (equation 8) centered on bacterial MIC times an estimate of 5.90 (ΔOFV = −117.20, for two additional parameters). The release of endotoxin from filaments is described by equations 9 and 10.

### Final model and predictions.

**FIG 3:**
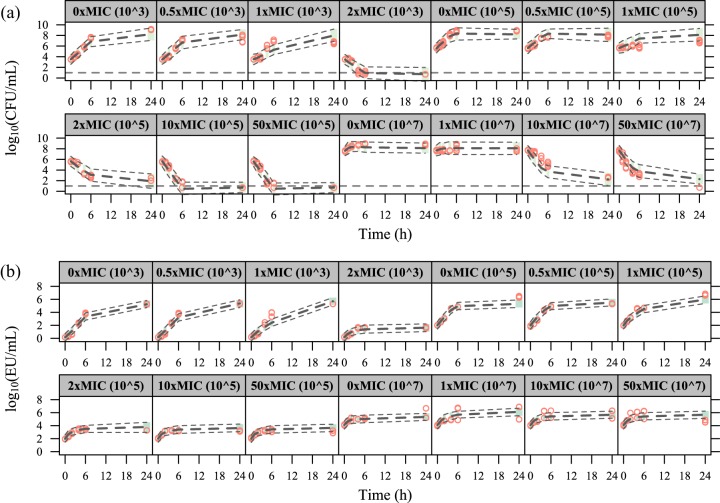
Visual predictive checks for the time courses of CFU (a) and endotoxin units (EU) (b) in the experiments with static cefuroxime concentrations. The red circles correspond to the observed data, while the long-dashed lines correspond to the median simulated data. The shaded area between the two short-dashed lines corresponds to the 95% confidence interval (CI) around the simulated median data. The horizontal dashed line in panel a indicates the lower limit of detection (10 CFU/ml).

**FIG 4:**
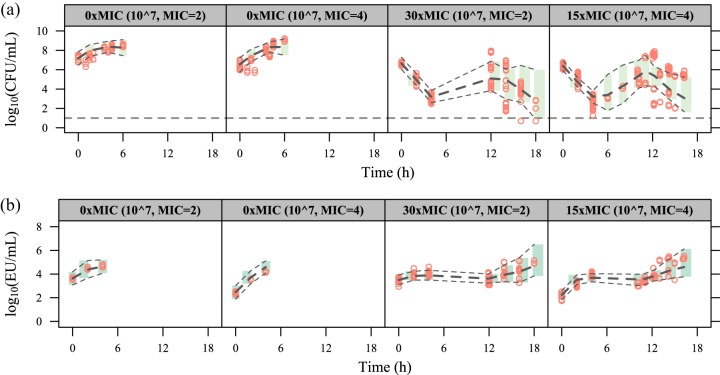
Visual predictive checks for the time courses of CFU (a) and endotoxin units (b) in the experiments with dynamic cefuroxime concentrations. The red circles correspond to the observed data, while the long-dashed lines correspond to the median simulated data. The shaded area between the two short-dashed lines corresponds to the 95% CI around the simulated median data. The horizontal dashed line in panel a indicates the lower limit of detection (10 CFU/ml).

A delay in endotoxin release following antibiotic-induced killing of susceptible and filamentous bacteria (one transit compartment with a mean transit time of 7.63 h) significantly improved the fit (ΔOFV = −165.09). No improvement was observed with a delay of endotoxin release due to bacterial growth. The contribution to endotoxin release was as follows: 0.000292 endotoxin units (EU) per grown (*k_G_*_,_*_ETX_*) CFU, 0.00636 EU per killed susceptible (*k_K_*_,_*_ETX_*) CFU, and 0.295 EU per killed filamentous (*k_F_*_,_*_ETX_*) CFU. Parameter estimates and their uncertainty are presented in Table 1, and model fits for CFU and endotoxin time courses are shown in Fig. 3 for static cefuroxime concentrations and in Fig. 4 for dynamic cefuroxime concentrations.

**TABLE 1:** Overview of parameters in the final model with values estimated on all data (including both static and dynamic cefuroxime concentrations)^*a*^

Parameter (unit)	Parameter description	Estimate (RSE [%])
*k*_growth_ (h^−1^)	Bacterial growth rate	1.45 (5.1)
*k*_death_ (h^−1^)	Bacterial natural death rate	0.179 (fix)
*B*_max_ (CFU/ml)	Bacterial system capacity	1.42 × 10^8^ (19)
*T_SR_* (CFU/ml)	Threshold for transition from *S* state to *R* state	7.01 × 10^7^ (14)
*E*_max_ (h^−1^)	Maximum antibiotic-induced killing rate	3.26 (3.8)
EC_50_ (mg/liter)	Potency for drug effect (MIC dependent)	
	MIC = 2 mg/liter	2.93 (2.7)
	MIC = 4 mg/liter	4.88 (11)
γ	Sigmoidal Hill coefficient	3.37 (25)
*k_deg_* (h^−1^)	Degradation rate of antibiotic (both systems)	0.026 (fix)
*k_el_* (h^−1^)	Elimination rate (dynamic system)	0.462 (fix)
Mix	Fraction of experiments with typical elimination	0.574 (24)
Offset	Proportional change for atypical elimination	1.36 (4.7)
*k_G_*_,_*_ETX_* (EU/CFU)	LPS/endotoxin from bacterial growth (*S*)	0.000292 (11)
*k_K_*_,_*_ETX_* (EU/CFU)	LPS/endotoxin from antibiotic-killed bacteria (*S*)	0.00636 (25)
*k_FK_*_,_*_ETX_* (EU/CFU)	LPS/endotoxin from antibiotic-killed filaments (*F*)	0.295 (43)
*k_tr_*_,_*_ETX_* (h^−1^)	Transit rate for LPS/endotoxin release (*S* + *F*)	0.131 (27)
θ_MIC,_*_F_*	Concentration × MIC for filament formation	5.90 (8.2)
ω^2^	CFU unexplained residual variance	0.409 (12)
ω_repl_^2^	CFU replication error (plate spread) variance	0.0363 (16)
ω^2^	LPS/endotoxin unexplained residual variance	0.164 (13)

a EU, endotoxin units; *F*, filament biomass state; *R*, resting state; RSE, relative standard error; *S*, susceptible state.

**FIG 5:**
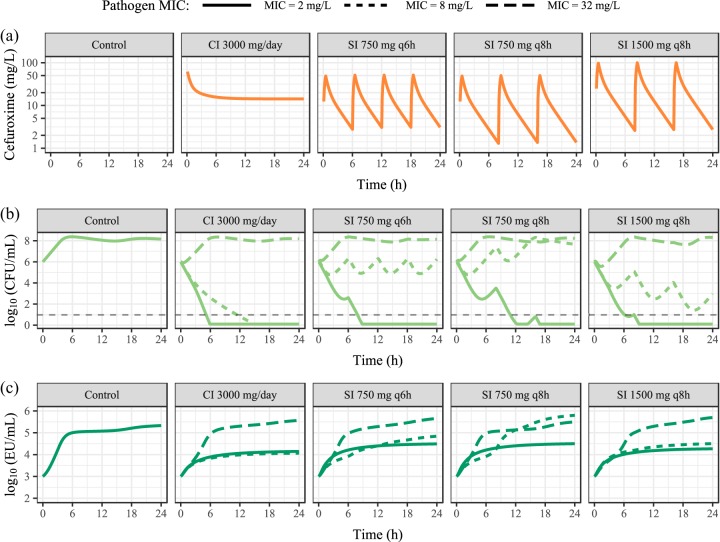
Prediction from the final model of LPS/endotoxin release under various cefuroxime treatment regimens during infection of pathogens with three MIC values (2, 8, and 32 mg/liter) (shown in the key) at an inoculum of 10^6^ CFU/ml. Treatment regimens shown are constant infusion (CI) preceded by a 4-min infusion of 750 mg and 30-min infusions of 750 mg given every 6 h (q6h) and 750 and 1,500 mg given every 8 h (q8h). The horizontal dashed line in panel b indicates the lower limit of detection (10 CFU/ml).

The treatment regimens used in the predictions resulted in typical cefuroxime exposure, CFU, and endotoxin release time courses, as shown in Fig. 5. When CFU are rapidly reduced, endotoxin concentrations reach approximately 10^4^ to 5 × 10^4^ EU/ml, much lower than the control levels of approximately 3 × 10^5^ EU/ml, even though regimens with a 750-mg dose show slightly higher endotoxin release than other doses due to less rapid killing. With increasing MICs, the lower cefuroxime-induced killing of bacteria leads to endotoxin release from bacterial growth and antibiotic-induced killing simultaneously, producing a higher endotoxin concentration of between approximately 10^5^ and 5 × 10^5^ EU/ml.

## DISCUSSION

The presented work establishes a new mathematical model that can be used for the exploration of endotoxin release in the context of antibiotic administration or bacterial growth, by the quantitative link of release to the two processes of bacterial growth and antibiotic-induced killing. Three E. coli strains exposed to the β-lactam antibiotic cefuroxime in setups with static or dynamic concentrations (24) and various inoculum sizes provided sufficient data (Fig. 1) to establish the novel model framework presented in Fig. 2.

The dynamics between cefuroxime and bacteria were described by a previously reported model structure (22, 23), with reasonable parameter estimates, in relation to both the data and previously reported values for the maximum antibiotic-induced killing rate (*E*_max_) (23), bacterial system capacity (*B*_max_) (23), and bacterial growth rate (*k*_growth_) (26). In addition to separate EC_50_ estimates for MIC values of 2 and 4 mg/liter (2.93 and 4.88 mg/liter, also parameterized so that EC_50_ estimates are proportional to the MIC) (26), a high γ of 3.37 was estimated, likely true for β-lactams where thresholds (e.g., *fT*_>MIC_ [time of a 24-h period that the drug concentration exceeds the MIC of the free, unbound fraction of the drug under steady-state pharmacokinetic conditions]) are often linked to the effect (27). Further adaptations were needed to fit the high-inoculum (10^7^ CFU/ml) and high-exposure (50× MIC) data, achieved with the inclusion of a step function (equation 1) to limit the transition of susceptible bacteria to the resting state at low bacterial counts. This is in line with work on persisters (represented by part of the resting state), which exhibit a sharp increase in the mid- to late exponential growth phase in E. coli (28). The threshold estimate of approximately 7 × 10^7^ CFU/ml may be considered high compared to the maximum system capacity of 1.42 × 10^8^ CFU/ml but considerably improved the fit of observed rapid kill at a high inoculum. Considerable variability in regrowth in the dynamic experiments has been reported previously (29) but may in part be attributed to experimental variability (such as pump performance, indicated in some dynamic experiments). The model described two subpopulations, with some experiments assigned a 36% increased (estimated) *k_el_*, with a strong probability of assignment (0.95 to 0.99) across dynamic experiments (30, 31). While not measured, the optimal solution would have been to develop a model for the cefuroxime concentrations (24).

The model that accounts for endotoxin concentrations was developed by quantitatively relating the generated and killed CFU, through processes of bacterial growth and antibiotic-induced killing, to endotoxin release, assuming that endotoxin did not degrade at 37°C during the experiments. Endotoxin is regarded as extremely heat stable (3, 32, 33), and with the constant concentrations observed between 6 and 24 h in the high-exposure experiments (10× and 50× MIC), in light of the rapid reduction in CFU, degradation under the reported experimental conditions is unlikely. Linking release to growth is sensible, as control experiments achieve high endotoxin concentrations after 24 h, and it is probable that the process of binary fission leads to shedding of endotoxins into the solution (34). The instantaneous endotoxin release due to bacterial growth may be explained as occurring only during the separation into two bacteria. The delayed and prolonged release following antibiotic-induced kill, reported previously to be between 0.5 and 6 h (35, 36), shows that endotoxin release from a single CFU following lysis is a delayed process.

A linear relationship between the log_10_ CFU reduction and log_10_ increase in endotoxin has previously been proposed (37). However, control experiments demonstrate that growing bacteria rapidly lead to an increase in endotoxin (0.000292 EU per new CFU), although the curve eventually plateaus as growth becomes limited due to the capacity of the system. Antibiotic-induced killing produces a larger release (0.00636 EU per killed CFU), which is expected and was shown previously for β-lactam antibiotics that exert bactericidal effects (36).

While not quantified in the present study, a previous assessment of similar β-lactam antibiotics (ceftazidime and cefotaxime) demonstrated that long filaments are formed at concentrations at and above the pathogen MICs for various E. coli isolates, including the ATCC 25922 reference strain used in the present study (21). The incorporated filament formation is predicted to occur more extensively in the dynamic experiments with less initial kill (15× MIC), as more bacteria are available for transition into the filamentous state at relevant concentrations in the dynamic cefuroxime concentration-time profile. As the formed filaments vary in length and, thus, endotoxin content (21), the estimated release of 0.295 EU per antibiotic-killed filament (46 times higher than an antibiotic-killed susceptible bacterium) is based on an empirical relation without clear mechanistic interpretation. Indeed, the estimate of 0.295 EU/CFU is negatively correlated with the number of filamentous bacteria, governed by the transition of bacteria from the susceptible state to the filamentous state, with an assumed peak of 1. While the assessment of different peak rates (0.5, 2, and 4) illustrated this, it is noteworthy that estimates of θ_MIC__-_*_F_*, *k_G_*_,_*_ETX_*, and *k_K_*_,_*_ETX_* remained close to the reported final values. However, extensive filament formation at clinically relevant concentrations for the treatment of intermediate or less susceptible bacteria (represented by high MICs of 8 and 32 mg/liter, which were not studied experimentally in the present work) (21) may be extended to an expectation of an increased release of endotoxins, as demonstrated in Fig. 5. It should be noted that the predictions are based on the extrapolation of EC_50_s estimated for relatively susceptible bacteria (MICs of 2 and 4 mg/liter) to high MICs, and even though a strong EC_50_-MIC correlation has been shown in previous studies (26, 38), the fact that less susceptible strains were not studied is a limitation. Nevertheless, increased release of endotoxins may be added to the list of hazards facing patients suffering from infections with intermediate or less susceptible bacteria.

Even though a single bacterial species (E. coli) and antibiotic (cefuroxime) were used, the structural model obtained in this work could be generalized and applied to other Gram-negative bacteria and antibiotics of the β-lactam class. While the point estimates for endotoxin release per CFU and the relative contribution of filaments to overall release are expected to depend strongly on the drug-bug PBP specificity (17, 33) and, thus, vary within and between antibiotic classes, the overall findings are expected to be generalizable for β-lactams and Gram-negative species.

Although preclinical relations between antibiotic-induced endotoxin release and mortality have been established (39), this connection has been difficult to observe in clinical practice. Problematic assaying of endotoxin and the diagnostic relevance of endotoxemia in the clinic, discussed previously (40, 41), represent possible causes of these difficulties. Complexities such as variability in endotoxin heterogeneity, content across strains, and the release of other associated bacterial products add to the problem, in addition to underlying patient heterogeneity (infecting organisms, comorbidities, and immune status, etc.).

While the use of three strains adds to the generalizability of the established model, examining one strain under both static and dynamic setups would have added value by allowing examination of the potential system effects on both growth and endotoxin release. The developed model could be informed by new static experiments with less susceptible bacteria or with cefuroxime concentrations in the range predicted to induce filament formation, in addition to quantification of the number of produced filaments. An additional aspect would be the inclusion of data from combination treatment, as the addition of an aminoglycoside with the second cefuroxime dose has been shown to reduce the release of endotoxin (24), with less visible filament formation due to inhibition of protein synthesis. Antibiotic-induced endotoxin release has also been studied in the context of bacteriophage therapy, with observations of increased survival (42) and diminished endotoxin release (43) compared to conventional antibiotics and controls. The addition of such data with a different interaction between “drug” and bacteria would provide an extended model.

In conclusion, processes of bacterial growth and antibiotic-induced bacterial killing were successfully tied to endotoxin release and quantified in a mathematical model. Experiments with observed (but not quantified) filament formation were incorporated and recognized as having increased endotoxin release upon kill. Taken together, endotoxin release over 24 h is lowest when antibiotic exposure rapidly eradicates the bacteria and highest at doses that lead to fluctuating concentrations around the MIC, allowing for simultaneous division, drug-induced kill, and, potentially, filament formation.

## MATERIALS AND METHODS

Time-kill experiments with static cefuroxime concentrations were designed for the purpose of this study and complemented with previously reported time-kill experiments using dynamic concentrations (24).

### *In vitro* experiments.

Test strains were inoculated in pyrogen-free glass tubes containing brain heart infusion broth prior to experiments and incubated for 4.5 h at 35°C to bring the bacteria into the logarithmic growth phase.

In the time-kill experiments with static antibiotic concentrations, a clinical E. coli strain (B09-118-22; MIC of 2 mg/liter) was exposed to six cefuroxime concentrations (0×, 0.5×, 1×, 2×, 10×, and 50× MIC) across three starting inocula (approximately 10^3^, 10^5^, and 10^7^ CFU/ml). Experiments were performed in duplicate in brain heart infusion broth at 37°C, with samples for CFU counts and endotoxin analysis drawn before and 1, 2, 4, 6, and 24 h after the addition of the antibiotic.

The study with dynamic antibiotic concentrations, described previously (24), involved a clinical E. coli strain (B049-3036; MIC of 2 mg/liter) and a reference E. coli strain (ATCC 25922; MIC of 4 mg/liter). Experiments were repeated six and eight times for each strain (durations of 14 to 18 h), in addition to controls (durations of 4 to 6 h), and were performed at 37°C in a kinetic system (29) consisting of a spinner flask fitted with a pump and filter membrane (0.45 μm) to prevent the elimination of bacteria. Bacteria were added to the system at 0 h (starting inoculum of approximately 10^6^ CFU/ml), followed by cefuroxime at 0 h and at approximately 12 h to achieve a peak concentration of 60 mg/liter (15× or 30× MIC). The pump was configured to yield a cefuroxime half-life (*t*_1/2_) of 1.5 h (*k_el_* of 0.462 h^−1^), akin to that in patients (24, 44). Samples for CFU counts and endotoxin analysis were taken before and 2 and 4 h after the addition of the antibiotic.

For both experimental setups, samples were seeded onto at least two Columbia agar plates and incubated at 35°C for 48 h before viable counting of the bacteria (with a limit of detection [LOD] of 10 CFU/ml). For quantification of endotoxin, measured in endotoxin units (EU) per milliliter, samples were filtered and frozen at −70°C before being analyzed in duplicate with the kinetic chromogenic *Limulus* amebocyte lysate (LAL) assay (45).

### Software and model selection.

The generated data were analyzed in NONMEM 7.4.3 (46), using the Laplacian estimation method to obtain maximum likelihood estimates and incorporate CFU observations below the LOD (10 CFU/ml) by the use of M3 (47). For CFU, all replicate plate counts were included in the modeling, and L2 functionality was used to split CFU residual errors into sample- and replicate-specific errors (23). Additive residual errors to the log_10_ of predictions were used for both CFU counts and endotoxin samples.

Comparisons between two competing nested models were done using the likelihood ratio test based on the OFVs, a statistical measure to judge model fit, which is assumed to be χ^2^ distributed and where a ΔOFV of −3.84 is significant at a *P* value of 0.05 for one additional parameter. Additional diagnostics consisted of residual goodness of fit, simulation-based visual predictive checks (VPCs) (31, 48), and reasonable parameter estimates.

### Modeling of bacterial dynamics.

Modeling of bacterial dynamics started from a previously reported model describing Streptococcus pyogenes and cefuroxime in a static *in vitro* system (23). The model consists of differential equations describing antibiotic concentration (*C_AB_*), susceptible bacteria (*S*), and resting bacteria (*R*), with the model prediction of CFU per milliliter equal to the sum of *S* and *R*, as shown in the following equations:

(2) $$\frac{dC_{\mathrm{AB}}}{\mathrm{dt}}=-(k_{\deg}+k_{\mathrm{el}})\times C_{\mathrm{AB}}$$

(3) $$\frac{\mathrm{dS}}{\mathrm{dt}}=-k_{\text{death}}\times S-k_{\mathrm{SR}}\times S+k_{\mathrm{RS}}\times R+k_{\text{growth}}\times S-\left(\frac{E_{\text{max}}\times C_{\mathrm{AB}}{}^{\text{γ}}}{{\text{EC}}_{50}{}^{\text{γ}}+C_{\mathrm{AB}}{}^{\text{γ}}}\right)\times S$$

(4) $$\frac{\mathrm{dR}}{\mathrm{dt}}=-k_{\text{death}}\times R+k_{\mathrm{SR}}\times S-k_{\mathrm{RS}}\times R$$

where *k_deg_*, *k_el_*, *k*_death_, and *k*_growth_ are first-order rate constants describing antibiotic degradation, antibiotic elimination, natural bacterial death, and bacterial growth, respectively, and *k_SR_* describes the rate at which bacteria transform from the growing drug-susceptible state (*S*) into the resting state (*R*). The rate depends on total viable bacteria in the system (*S* + *R*) and is parameterized as (*k*_growth_ − *k*_death_)/*B*_max_ × (*S* + *R*), where *B*_max_ represents the system capacity. With this model implementation, the resting state of the bacteria is included in order to describe the observed biphasic killing behavior and inoculum effect. Parameters related to antibiotic effect refer to the maximum drug-induced killing rate (*E*_max_), potency as the concentration for a half-maximum killing rate (EC_50_), and the Hill coefficient (γ). Previously reported values were used and fixed for *k*_death_ (0.179 h^−1^), *k_RS_* (0 h^−1^), and *k_deg_* (0.026 h^−1^) (23).

For the study using dynamic cefuroxime concentrations, the pump was configured to simulate a *k_el_* of 0.462 h^−1^, which, after the addition of *k_deg_*, results in a final simulated half-life of 1.42 h. The inclusion of strains with various MICs led to an assessment of specific fixed effects for potency (EC_50_). Variable bacterial regrowth was observed following washout of the first cefuroxime dose and may be attributed to experimental variability (clogging and/or leakage affecting the *k_el_* of the drug from the system). This was assessed with a mixture model, which can describe two or more discrete populations with the likelihood determining the most probable *k_el_* for each experiment (30).

Adaptations such as a delay in bacterial growth and/or antibiotic-induced kill, correlation between bacterial MIC and EC_50_, or application of a threshold value for allowing the transition of susceptible bacteria into the resting bacterial state were also assessed.

### Modeling of endotoxin release.

The bacterial model was extended to endotoxin samples from experiments with static cefuroxime concentrations, with liberated endotoxin related to bacterial processes of (i) binary fission (growth [*G*]), (ii) antibiotic-induced killing (*K*), and (iii) natural bacterial death (*D*), described in the following equations:

(5) $$\frac{\mathrm{dG}}{\mathrm{dt}}=k_{G,\mathrm{ETX}}\times k_{\text{growth}}\times S-k_{\mathrm{el}}\times G$$

(6) $$\frac{\mathrm{dK}}{\mathrm{dt}}=k_{K,\mathrm{ETX}}\times \left(\frac{E_{\text{max}}\times C_{\mathrm{AB}}{}^{\text{γ}}}{{\text{EC}}_{50}{}^{\text{γ}}+C_{\mathrm{AB}}{}^{\text{γ}}}\right)\times S-k_{\mathrm{el}}\times K$$

(7) $$\frac{\mathrm{dD}}{\mathrm{dt}}=k_{D,\mathrm{ETX}}\times k_{\text{death}}\times (S+R)-k_{\mathrm{el}}\times D$$

where the release of endotoxin from the respective population of CFU was scaled by the constants *k_G_*_,_*_ETX_*, *k_K_*_,_*_ETX_*, and *k_D_*_,_*_ETX_* (representing EU per CFU). Endotoxin was assumed to be stable during the experimental duration (i.e., no degradation), with *k_el_* representing the elimination rate (set to zero in static experiments).

In the dynamic *in vitro* model, extensive formation of bacterial filaments was reported following the second antibiotic dose, accompanied by a large increase in the endotoxin concentration (24). The dynamic cefuroxime profile was assumed to include concentrations at which the affinity for PBP-3 led to bacterial elongation (i.e., constant CFU but increased biomass). Upon the administration of the second dose, elevated concentrations and affinity for PBP-1 then liberated an increased amount of endotoxin from the filaments (19). A Gaussian function (describing a bell-shaped curve) was assessed as follows:

(8) $$k_{\text{filament }}\left[f\left(C_{\mathrm{AB}}\right)\right]=e^{-\frac{{\left(C_{\mathrm{AB}}-{\text{θ}}_{\text{MIC},F}\times \text{MIC}\right)}^{2}}{2\times 1^{2}}}$$

where the first-order rate constant *k*_filament_, ranging from 0 to 1, facilitates the transition from the susceptible bacterial state (*S*) to a filamentous state (*F*), centered around an antibiotic concentration given by the pathogen MIC times an estimated parameter, θ_MIC,_*_F_*, described according to

(9) $$\frac{\mathrm{dF}}{\mathrm{dt}}=-\left(\frac{E_{\text{max}}\times C_{\mathrm{AB}}{}^{\text{γ}}}{{\text{EC}}_{50}{}^{\text{γ}}+C_{\mathrm{AB}}{}^{\text{γ}}}\right)\times F-k_{\text{death}}\times F+k_{\text{filament}}\times S+k_{\text{growth}}\times F$$

(10) $$\frac{\mathrm{dFK}}{\mathrm{dt}}=k_{F,\mathrm{ETX}}\times \left(\frac{E_{\text{max}}\times C_{\mathrm{AB}}{}^{\text{γ}}}{{\text{EC}}_{50}{}^{\text{γ}}+C_{\mathrm{AB}}{}^{\text{γ}}}\right)\times F-k_{\mathrm{el},\mathrm{ETX}}\times \mathrm{FK}$$

where equation 9 describes the formation of filaments. The additional growth in *F* (*k*_growth_, the same value as in equation 3 for *S*) would not contribute to the observed bacterial counts (*S* + *R* + *F*) but represents an increase in biomass, which leads to increased endotoxin release upon antibiotic-induced killing of the filaments. Similar to equations 5 to 7, the number of EU released per killed filament (described by *k_F_*_,_*_ETX_*) is shown in equation 10.

Taken together, the model prediction of EU per milliliter becomes the sum of the endotoxin concentration observed at baseline and bacterial endotoxin release from growth (*G*), antibiotic-induced killing of susceptible bacteria (*K*), natural death (*D*), and antibiotic-induced killing of filaments (*FK*).

Based on the final model, endotoxin release for an assumed infection (49) of 10^6^ CFU/ml and the corresponding *in vitro* endotoxin baseline were predicted by utilizing a population pharmacokinetic model for cefuroxime (44) and assuming an unbound fraction of 0.67 (50). As estimates of endotoxin clearance are not available for humans, *k_el_* was set to zero to illustrate cumulative endotoxin release. Clinical regimens were chosen based on Summary of Product Characteristics (SmPC) recommendations (51) and consisted of a continuous infusion of 3,000 mg/24 h preceded by a 4-min infusion of 750 mg, a 30-min infusion of 750 mg given every 6 h, and 30-min infusions of 750 or 1,500 mg given every 8 h. Three MICs (2, 8, and 32 mg/liter) were used to illustrate endotoxin release under various combinations of antibiotic-induced killing and microbial growth, with EC_50_s scaled proportionally to the MIC.
